# Establishment of the Prognostic Index Reflecting Tumor Immune Microenvironment of Lung Adenocarcinoma Based on Metabolism-Related Genes

**DOI:** 10.7150/jca.49266

**Published:** 2020-10-18

**Authors:** Jianguo Zhang, Jianzhong Zhang, Cheng Yuan, Yuan Luo, Yangyi Li, Panpan Dai, Wenjie Sun, Nannan Zhang, Jiangbo Ren, Junhong Zhang, Yan Gong, Conghua Xie

**Affiliations:** 1 Department of Radiation and Medical Oncology, Zhongnan Hospital of Wuhan University, Wuhan, Hubei 430071, China; 2 Department of Occupational and Environmental Health, School of Public Health, Qingdao University, Shandong 266021, China; 3 Department of Biological Repositories, Zhongnan Hospital of Wuhan University, Wuhan, Hubei 430071, China; 4 Hubei Key Laboratory of Tumour Biological Behaviors, Zhongnan Hospital of Wuhan University, Wuhan, Hubei 430071, China; 5 Hubei Cancer Clinical Study Center, Zhongnan Hospital of Wuhan University, Wuhan, Hubei 430071, China.

**Keywords:** lung adenocarcinoma, metabolic landscape, prognostic index, bioinformatics, tumor immune microenvironment

## Abstract

**Background:** The incidence of lung adenocarcinoma (LUAD) increased substantially in recent years. A systematic investigation of the metabolic genomics pattern is critical to improve the treatment and prognosis of LUAD. This study aimed to analyze the relationship between tumor microenvironment (TME) and metabolism-related genes of LUAD.

**Methods:** The data was extracted from TCGA and GEO datasets. The metabolism-related gene expression profile and the corresponding clinical data of LUAD patients were then integrated. The survival-related genes were screened out using univariate COX regression and lasso regression analysis. The latent properties and molecular mechanisms of these LUAD-specific metabolism-related genes were investigated by computational biology.

**Results:** A novel prognostic model was established based on 8 metabolism-related genes, including TYMS, ALDH2, PKM, GNPNAT1, LDHA, ENTPD2, NT5E, and MAOB. The immune infiltration of LUAD was also analyzed using CIBERSORT algorithms and TIMER database. In addition, the high- and low-risk groups exhibited distinct layout modes in the principal component analysis.

**Conclusions:** In summary, our studies identified clinically significant metabolism-related genes, which were potential signature for LUAD diagnosis, monitoring, and prognosis.

## Introduction

Lung cancer as one of the tumors with high prevalence, leads to 1.7 million deaths worldwide annually 1. The deaths of lung cancer are more than the sum of the breast, colorectal and cervical cancers 2. Non-small cell lung cancer (NSCLC) accounts roughly for 85% lung cancer cases 3, and lung adenocarcinoma (LUAD) accounts for approximately 50% of NSCLC 4. Although the technologies in early detection, targeted therapy, and chemotherapy were substantially improved during last decades, the overall survival (OS) of LUAD patients remains poor 5. The research of target genes through RNA expression profiles became a hot topic in the prognosis of LUAD patients recently 6. Identification of metabolism-related genes is urgent and highly required to improve clinical outcomes of LUAD.

Cell metabolism is central to the survival and development of cells. Tumor cells have abnormal cell metabolism owing to the loss function of tumor suppressor genes or the activation of oncogenes. Increased glycolysis in tumor cells, manifested by increased glucose acquisition and lactic acid synthesis, is an important feature, called the Warburg effect 7, 8. It provided tumor cells with more selective advantages with limited resources 9. Meanwhile, aerobic glycolysis produces fewer reactive oxygen species (ROS), which enabled cells to better resist oxidative stress and adapted to hypoxic environments 10. Different cancers had different metabolic phenotypes 11. Lactic acid was metabolized in lung tumors and contributed more to tricarboxylic acid cycle than glucose 12. An animal study illustrated that inhibition of lactate dehydrogenase-A (LDH-A) controlled tumor survival and proliferation, as a feasible therapeutic target 13. The study of metabolism-related phenotypes of different cancers provided clues to the diagnosis and treatment of tumors. The metabolism of tumor cells would not only affect the proliferation, but also influence survival. Moreover, the alterations in TME resulted from the metabolic changes in tumor cells affected the metabolic levels and the activity of immune cells 14, 15. Therefore, regulating the metabolism of tumor cells and enhancing the activity of immune cells are important directions for the treatment of tumors.

We combined clinical information with metabolism-related genes expression profiles to evaluate the OS of LUAD patients. The prognostic landscape and expression status of metabolism-related genes were systematically analyzed, and individual prognostic characteristics for patients with LUAD were developed. We identified 8 metabolism-related genes significantly correlated with prognosis, and established a novel independent prognostic model based on these genes. This model also well predicted the infiltration of immune cells in LUAD. Our study provided a potential model and biomarkers for further metabolism-related work and personalized medicine for LUAD treatment.

## Materials and Methods

### Data collection and processing

**Figure 1:**
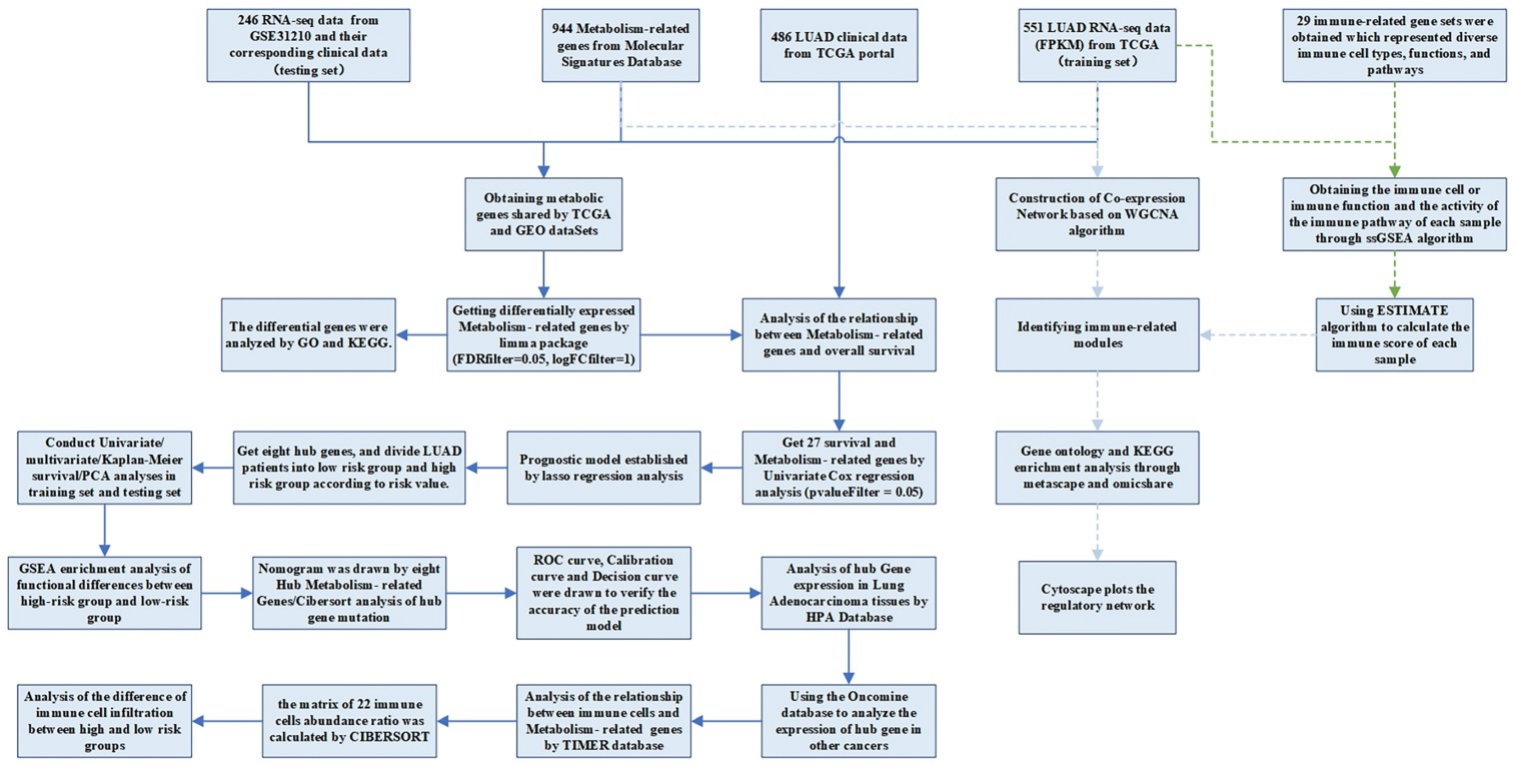
Flow chart of data processing in this study.

The RNA-seq FPKM data of LUAD, containing corresponding clinical data, were downloaded from the TCGA, including 497 LUAD tissues and 54 normal tissues. Patients whose follow-up data were incomplete or followed for less than 30 days were excluded, because these patients might die of non-tumor factors 16. A total of 454 patients were included in the following investigation. The demographic and clinical characteristics of the patient were listed in Table 1. The criteria of validation set selection were as follows: (1) Lung adenocarcinoma related; (2) Including complete follow-up data; (3) Sample size greater than 30; (4) Expression profiling by array; (5) Homo sapiens. The dataset (GSE31210) on NSCLC with survival data was also downloaded from the GEO database as a validation set. This dataset contained 20 normal samples and 226 tumor samples. We obtained the KEGG pathway gene sets from the Molecular Signatures Database and extracted all the metabolism-related genes. There were 863 metabolism-related genes shared by GEO and TCGA datasets. The analysis processes were shown in Figure 1.

### Differential expression analysis

To obtain differential metabolism-related genes in TCGA LUAD dataset, the limma package of R software was used to explore the genes in LUAD and its adjacent normal tissues. The log2 (fold-change) > 1 and false discovery rate (FDR) < 0. 05 were set as the cut-off values.

### Gene ontology and KEGG pathway analysis

To verify whether the differentially expressed genes were related to metabolism, GO and KEGG enrichment analysis were used. First, the org.Hs.eg.db package was used to convert the gene symbol into entrezID. Then, GO and KEGG enrichment analysis were performed using the clusterProfiler package. *P* < 0.05 was considered as statistical significance. Finally, the GOplot package was used to draw the circle diagram of GO and KEGG.

### Univariate COX analysis and LASSO analysis

To get survival- and metabolism-related genes, we integrated the expression of metabolism-related genes with the OS of LUAD patients. Metabolism-related genes were then analyzed by univariate COX regression analysis with continuous variables (*P <* 0.05). These metabolism-related genes were integrated into least absolute shrinkage and selection operator (LASSO) regression, which was calculated by the glmnet package of R software with 1,000 runs. Finally, the prognostic model of LUAD was established based on the LASSO regression co-efficiency multiplied by expression data. The formula was as follows:

Risk score=αgene(a)×gene expression(a)+αgene(b)×gene expression(b)+⋯+αgene(n)×gene expression(n)

### Survival analysis

The survminer package of R software was used to apply the Kaplan-Meier curve to investigate the connection amid metabolism-related genes and prognosis. Univariate analysis and multivariate analysis were used to explore independent prognostic factors of LUAD patients. Survival ROC R Software package was used to calculate the area under the curve (AUC) to verify the manifestation of prognostic characteristics. In addition, we drew a nomogram including the clinical factors and risk scores. The calibration curve and decision curve were painted to illustrate the accurateness of this model in predicting the survival of LUAD patients.

### Validation of the metabolism-related genes

To investigate the expression of metabolism-related genes in distinct cancers, the oncomine database was utilized to analyze the expression levels of the hub gene in tumor tissues and normal tissues. The Human Protein Atlas database was used to verify the protein function of metabolism-related genes by immunohistochemistry. The correlations between metabolism-related genes and clinical factors were also analyzed.

### Analysis of the relationship between immune cell infiltration and metabolism-related genes

The numbers of tumor-infiltrating immune cells were analyzed and visualized by TIMER database. TIMER reanalyzed gene expression data to assess the infiltrating levels of 6 immune cell subtypes, including CD4+ T cells, B cells, CD8+ T cells, neutrophils, macrophages, and dendritic cells. Therefore, it could be utilized to confirm the connections between hub metabolism-related genes and immune cell infiltration. We downloaded the levels of immune infiltration in LUAD patients and calculated the connection of immune cell infiltration and metabolism-related genes.

### Analysis of the difference between the high- and low-risk patients

With the median PI value, the patients were classified into 2 groups (low- and high-risk). PCA was utilized to analyze the grouped samples and expression patterns, and GSEA was used to estimate distinct function phenotypes of the 2 groups. CIBERSORT software package was used to evaluate the proportion of 22 leukocyte subtypes. The perm was set to 1000. The samples with P < 0. 05 in the results of CIBERSORT analysis were delivered for further investigation.

### Single sample gene set enrichment analysis

We obtained 29 immune-related gene sets that represented diverse immune cell types, functions, and pathways from Yin et al 17. Then we calculated the immune-related enrichment scores, which contained immune cell or immune function and the activity of the immune pathway, of each sample through the ssGSEA algorithm. Based on the enrichment scores, we performed hierarchical clustering of LUAD. In addition, the ESTIMATE algorithm was used to calculate the immune scores, which allowed specific and sensitive differentiation of immune cells and calculated the ESTIMATE scores to represent the proportion of tumor-infiltrating lymphocytes (TILs) in tumor tissues.

### Weighted gene co-expression network analysis (WGCNA)

To obtain metabolism- and immune-related genes, the WGCNA algorithm was applied to build the co-expression network of gene module and immune scores through the "WGCNA" package. Due to the small number of differential metabolic genes, we selected all metabolism-related genes for WGCNA analysis. First, we clustered the samples, eliminated the outlier, processed the data, and matched the samples with the expression matrix. Then, we selected the appropriate soft threshold to build a scale-free network and analyzed the module partition to acquire gene co-expression modules. Through the dynamic tree-cutting algorithm, we used dissimilarity matrices to detect gene modules. To get moderate-sized modules, the least count of genes was limited to 30 and modules with similar expression patterns were merged. Finally, we calculated the correlation between modulus feature genes and immune scores. We extracted the modules most relevant to immunity for GO and KEGG analysis through metascape and omicshare and plots the regulatory network by Cytoscape.

## Results

### Acquisition of differentially expressed metabolism-related genes

**Figure 2:**
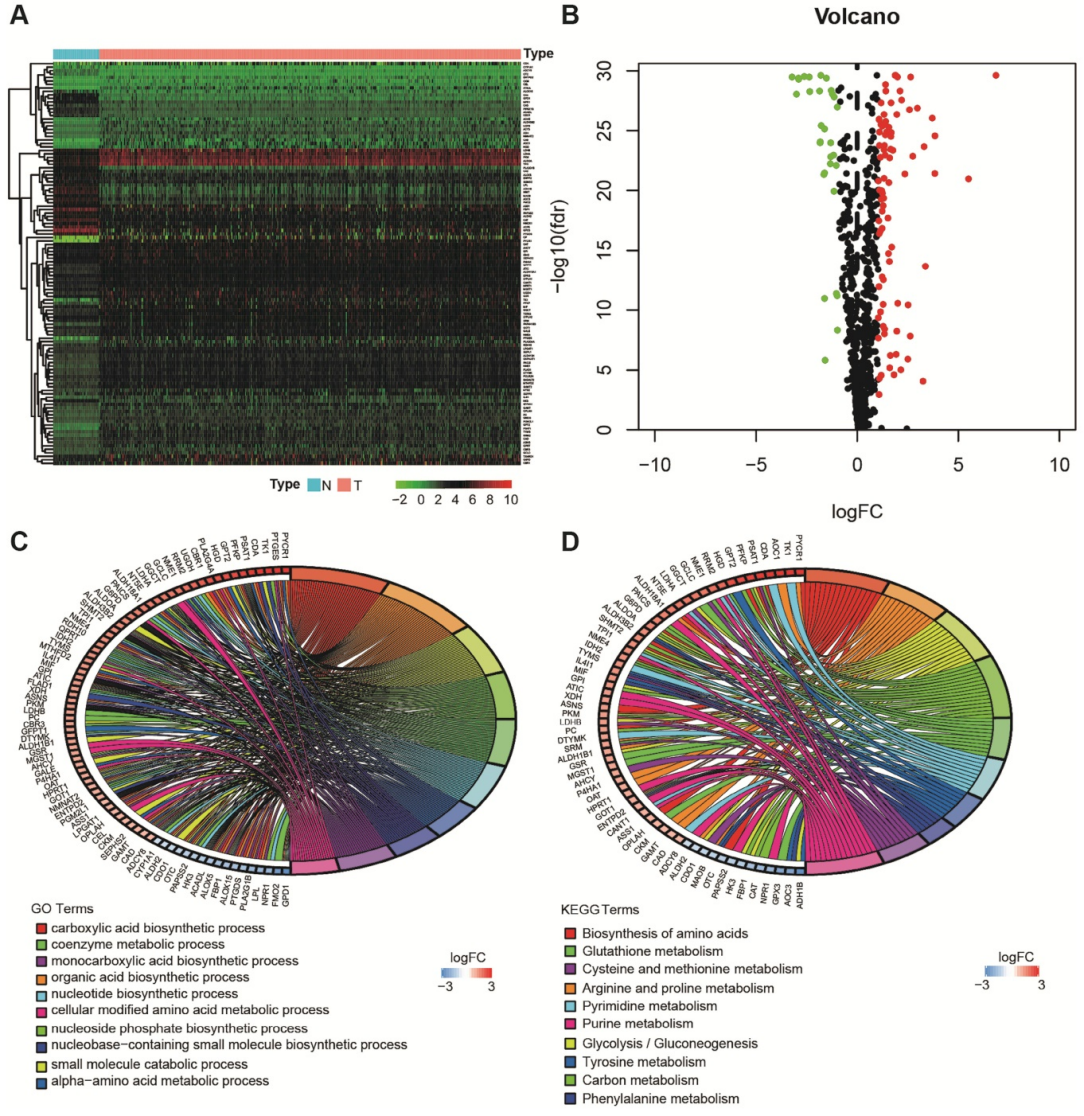
Acquisition of differentially expressed metabolism-related genes and gene functional enrichment analysis. **A**. Heatmap of differentially expressed genes. **B**. Volcano plot of differentially expressed genes. **C**. GO analysis. **D**. KEGG analysis.

Using the limma package of R software, the differences of 863 metabolism-related genes shared by TCGA and GEO were analyzed 18. We obtained 116 differentially expressed metabolism-related genes between LUAD tissues and neighboring normal tissues, containing 31 downregulated and 85 upregulated genes (Figure 2A and B). The results of GO analysis and KEGG analysis confirmed that the differential genes were related to metabolism (Figure 2C and D).

### Evaluation of clinical outcomes

**Figure 3:**
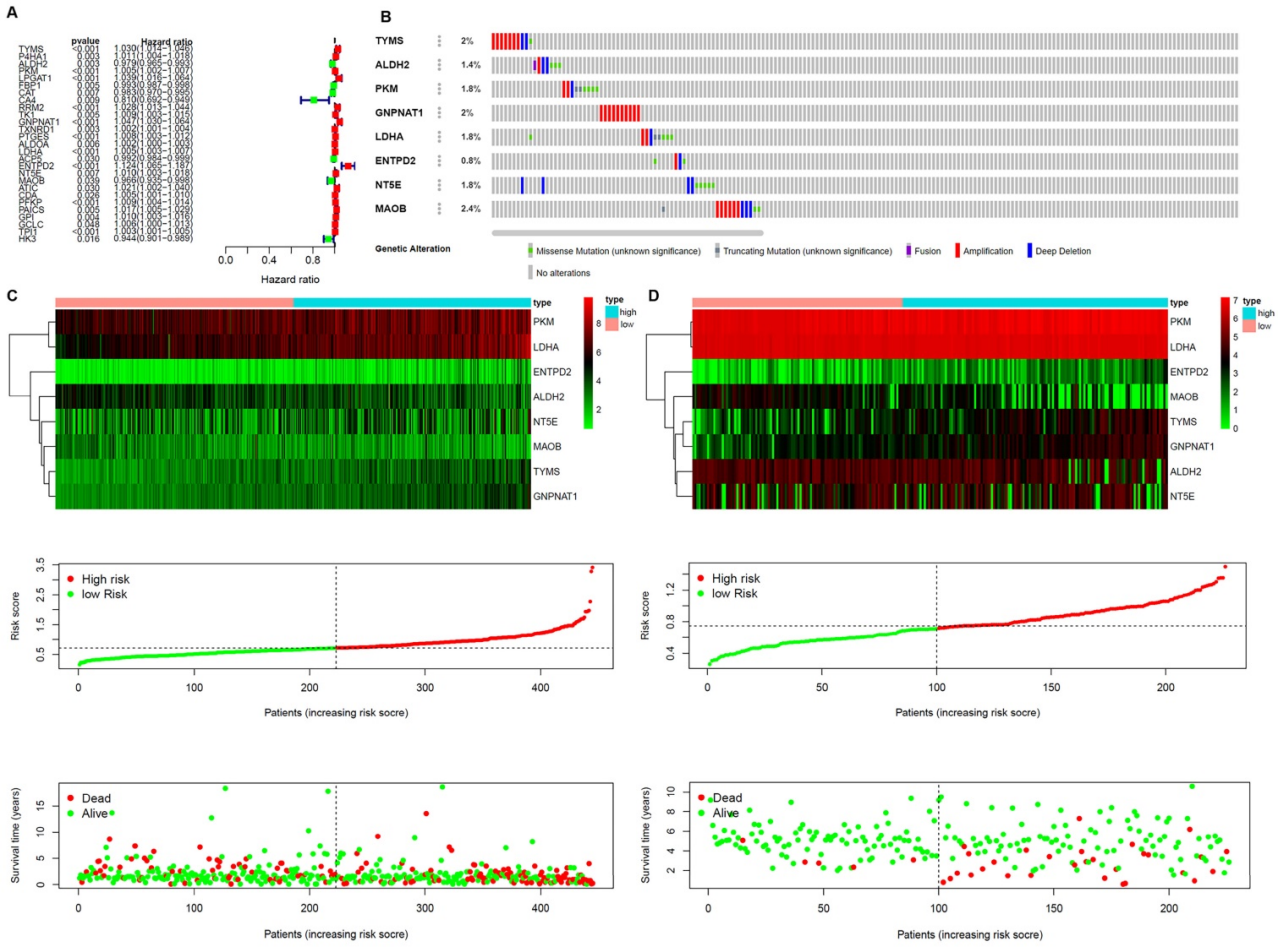
Establishment of Prognostic Indexes based on 8 metabolism-related genes **A**. Forest plot of hazard ratios exhibiting the prognostic worth of metabolism-related genes. **B**. Heatmap of expression profiles of included metabolism-related genes. Survival conditions of LUAD patients in **C**. training set and **D**. validation set.

A prognostic model was established based on these metabolism-related genes with univariate regression analysis and LASSO analysis. Through univariate COX regression analysis, 27 genes related to survival and metabolism (*P* value filter = 0.05) were obtained, of which the hazard ratio of 7 genes was less than 1 and the other 20 genes were greater than 1 (Figure 3A). Then we established the prognostic model by LASSO regression analysis. Eight metabolism-related genes were screened out to build the risk signature for LUAD (Table 2). The calculation formula of the risk score was shown as follows:

Risk score = [TYMS * (0.012119445)] + [ALDH2 * (-0.000559052)] + [PKM * (0.000501064)] + [GNPNAT1 * (0.022240778)] + [LDHA * (0.002514) + [ENTPD2 * (0.061360665)] + [NT5E * (0.00016529)] + [MAOB * (-0.004029927)]

**Figure 4:**
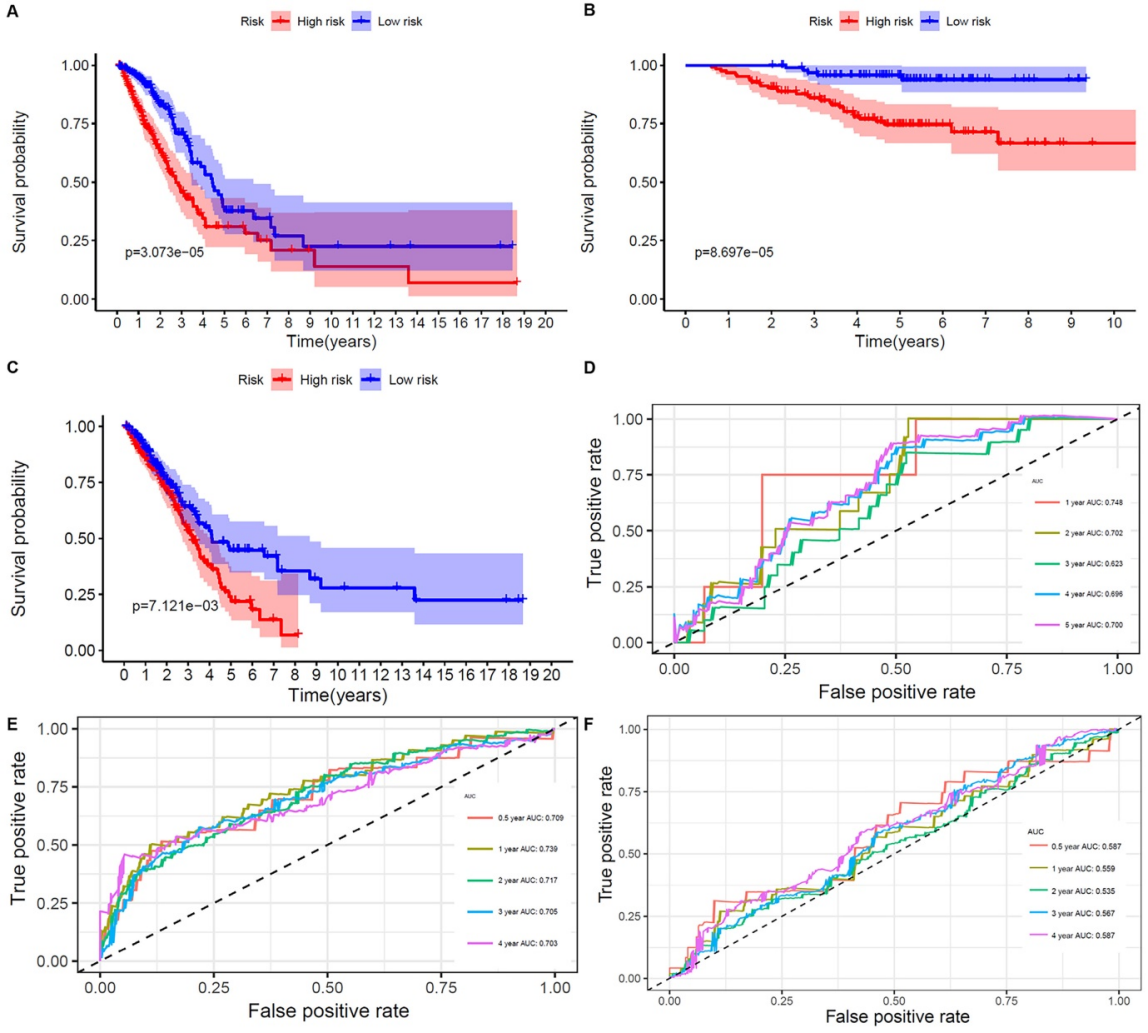
Overall survival of the low- and high-risk groups.** A**, **B**, and** C**. The prognostic worth of the biomarkers. Patients in the high‐risk groups sustained a shorter survival time in both the training set, validation set, and the other metabolism-related prognosis model. **D**. ROC curve verifies the accuracy of the model in predicting the 1-, 2- , 3- ,4-, 5-year survival rates of LUAD patients in the validation set. **E**. ROC curve verifies the accuracy of the model in predicting the 0.5-, 1-, 2- , 3- ,4-year survival rates of LUAD patients in the training set. **F**. ROC curve verifies the accuracy of the other model in predicting the 0.5-, 1-, 2-, 3-, and 4-years survival rates of LUAD patients.

The results showed that ALDH2 and MAOB were protective factors for OS, and the rest were risk factors. With the risk values, the patients were classified into 2 groups (low- and high-risk). In the genetic mutations of these risk genes, deep deletion and amplification were the most common forms (Figure 3B). MAOB had the most genetic alternations. The survival status, survival time and metabolism-related genes expression levels of the LUAD patients were shown in Figure 3C and D. With the increase in the risk scores, the numbers of deaths were also increased. The results of survival analysis showed that the clinical outcome of high- and low-risk groups was well distinguished according to these metabolism-related biomarkers in training and validation sets (Figure 4A and B). We compared our metabolism-related prognostic model with a similar model 19, 20. The result showed that the clinical outcome of high- and low-risk groups could also be well distinguished (Figure 4C). The AUC values of risk genes were 0.709, 0.739, 0.717, 0.705, 0.703 and 0.748, 0.702, 0.623, 0.696, 0.700 in training set, and validation set, respectively (Figure 4D and E). However, the AUC values of the other model were 0.587, 0.559, 0.535, 0.567, and 0.587, which illustrated that their model was less accurate in predicting the survival rate of LUAD patients compared with our model (Figure 4F).

### Analysis of subgroup and independent prognosis

The risk scores calculated by prognostic markers were helpful for the prediction of OS in different subgroups, containing stages I-II, stages III-Ⅳ, age > 60 years, age ≤ 60 years, N0-1, N2-3, T1-2 and T3-4 in the training set, and age > 60 years, age ≤ 60 years, female and male in the validation set (Figure S1).

The results of the univariate COX regression analysis showed the *P* values of stage and risk scores were less than 0.05 in the training and validation sets (Figure S2A and C). In addition, multivariate COX regression analysis verified that risk score (HR = 7.809; 95% CI [2.101-29.029]; *P* < 0.001; training set; HR = 4.361; 95% CI [1.020-28.642]; *P* < 0.001; validation set; Figure S2B and D) and stage (HR = 4.232, 95% CI [2.175-8.236]; *P* < 0.001; training set; HR = 3.405, 95% CI [1.690-6.862]; *P* < 0.001; validation set; Figure S2B and D) were independent risk factors in the training and validation sets. These results suggested that our signature could be utilized as an independent predictor for LUAD outcome.

### Clinic correlation and nomogram of metabolism-related genes

**Figure 5:**
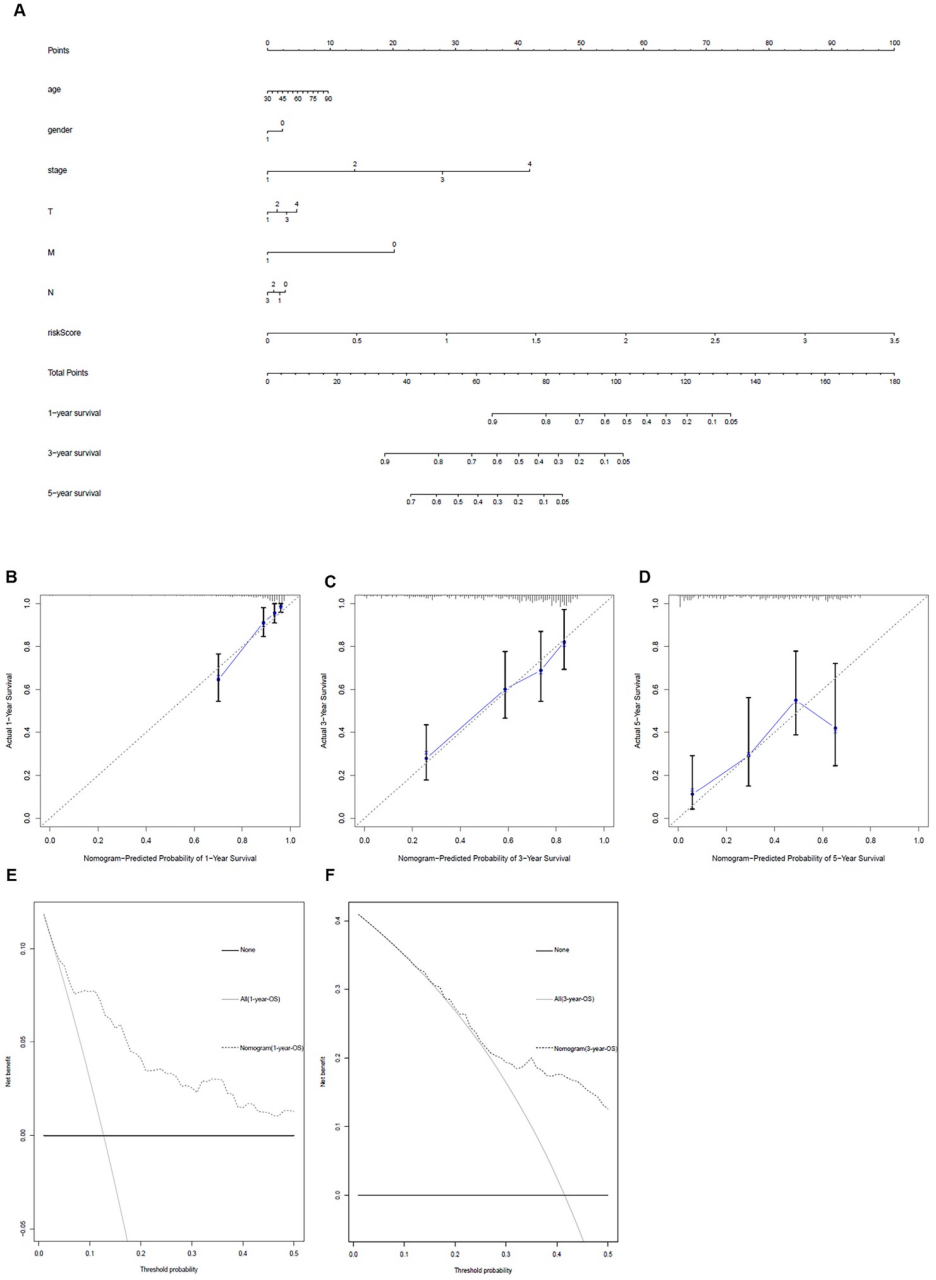
Metabolism-related genes combined with other clinical factors to predict the prognosis of patients with LUAD**. A**. Nomogram. **B**, **C** and** D**. The calibration curve was drawn to verify the accuracy of the prediction model for predicting 1-, 3-, and 5-years survival rates. **E** and **F**. Decision curve analysis of the nomogram.

The ggpubr package was applied to explore the connection of metabolism-related genes and clinical factors (Table 3). GNPNAT1, LDHA, MAOB, NT5E, PKM were associated with clinical factors. At the same time, we utilized metabolism-related genes together with clinical factors to draw a nomogram (Figure 5A) and the calibration curve was drawn to verify the accuracy of the prediction model (Figure 5B-D). The predicted value fit well with the real value, suggesting that our model might be applied to prophesy the prognosis of LUAD patients. DCA was performed to measure the clinical effectiveness of the nomogram. For the 1- and 3-years OS probability, the decision curve showed that the net benefits backed by the nomogram were better than those of the alternatives (Figure 5E and F).

### Validation of the metabolism-related genes

Based on the HPA database, the function of metabolism-related genes was verified at the protein levels by immunohistochemistry (Figure S3A). The results were accordant with our preceding research. Except for MAOB and ALDH2, the expression levels of other metabolism-related genes in LUAD was all in the tumor tissues. Oncomine analysis of tumor and normal tissues (Figure S3B) showed that the expression patterns of metabolism-related genes were similar in LUAD and other cancers.

### Immunocyte infiltration in the TME

**Figure 6:**
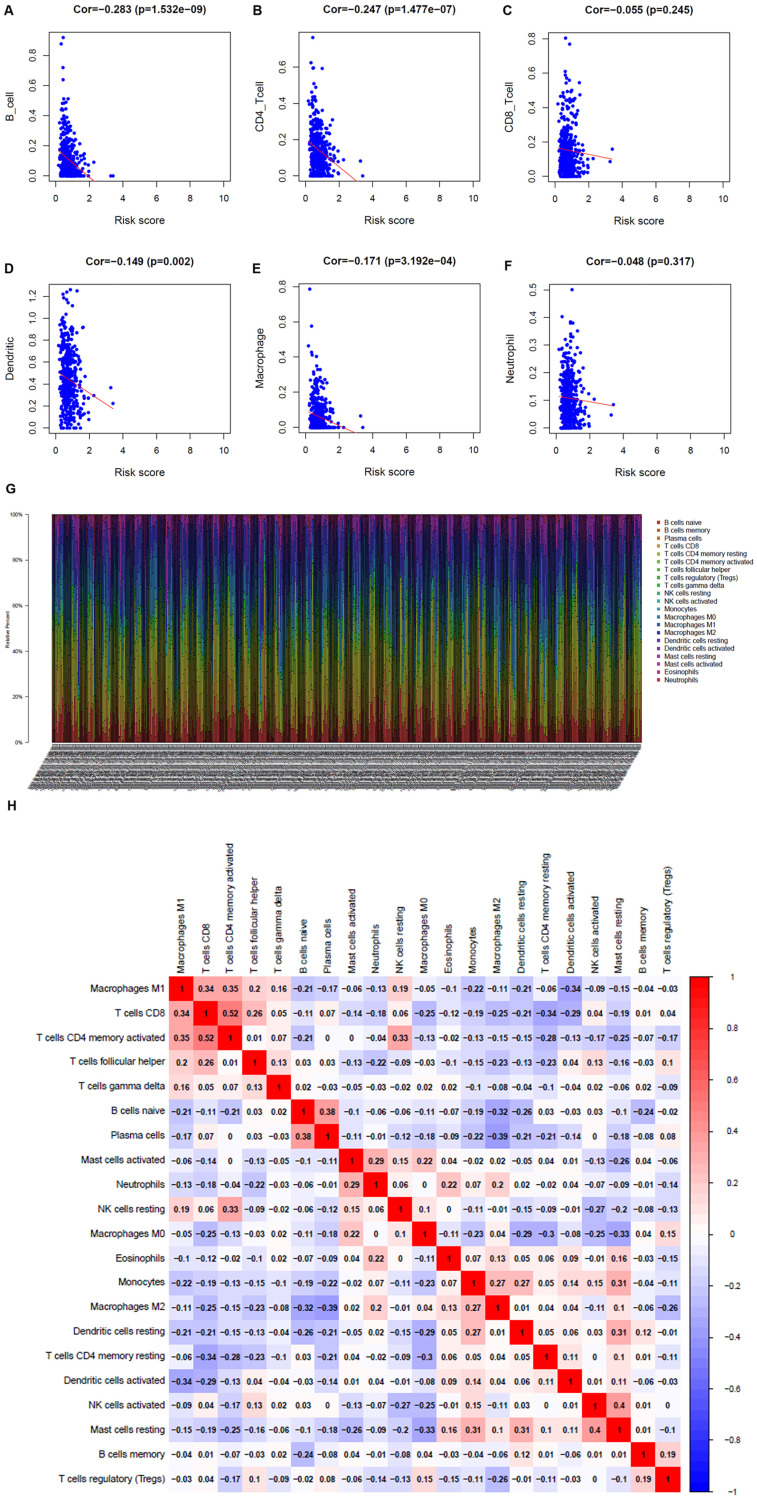
Analysis of the correlation between metabolism-related genes and immune cells. **A**. B cells. **B**. CD4+ T cells. **C**. CD8+ T cells. **D**. Dendritic cells. **E**. Macrophages. **F**. Neutrophils. **G**. The bar chart shows the proportion of immune cells in each patient. **H**. Correlation analysis of immune cells.

To understand whether the immune metabolic genome was related to the condition of the LUAD immune microenvironment, the TIMER database was applied to investigate the connection of the metabolism-related genes and immune cell infiltration. B cells, CD4+ T cells, dendritic cells, and macrophages were negatively related to metabolism-related genes (Figure 6A-F). The proportion of 22 immune cells in LUAD was shown in Figure 6G. Pearson correlation analysis illustrated the co-expression mode among different immune cells. There was a significant correlation between activated memory CD4+ T cells and CD8+ T cells, and a negative correlation between M2 macrophages and plasma cells (Figure 6H).

### Analysis of the difference between the high- and low-risk patients

**Figure 7:**
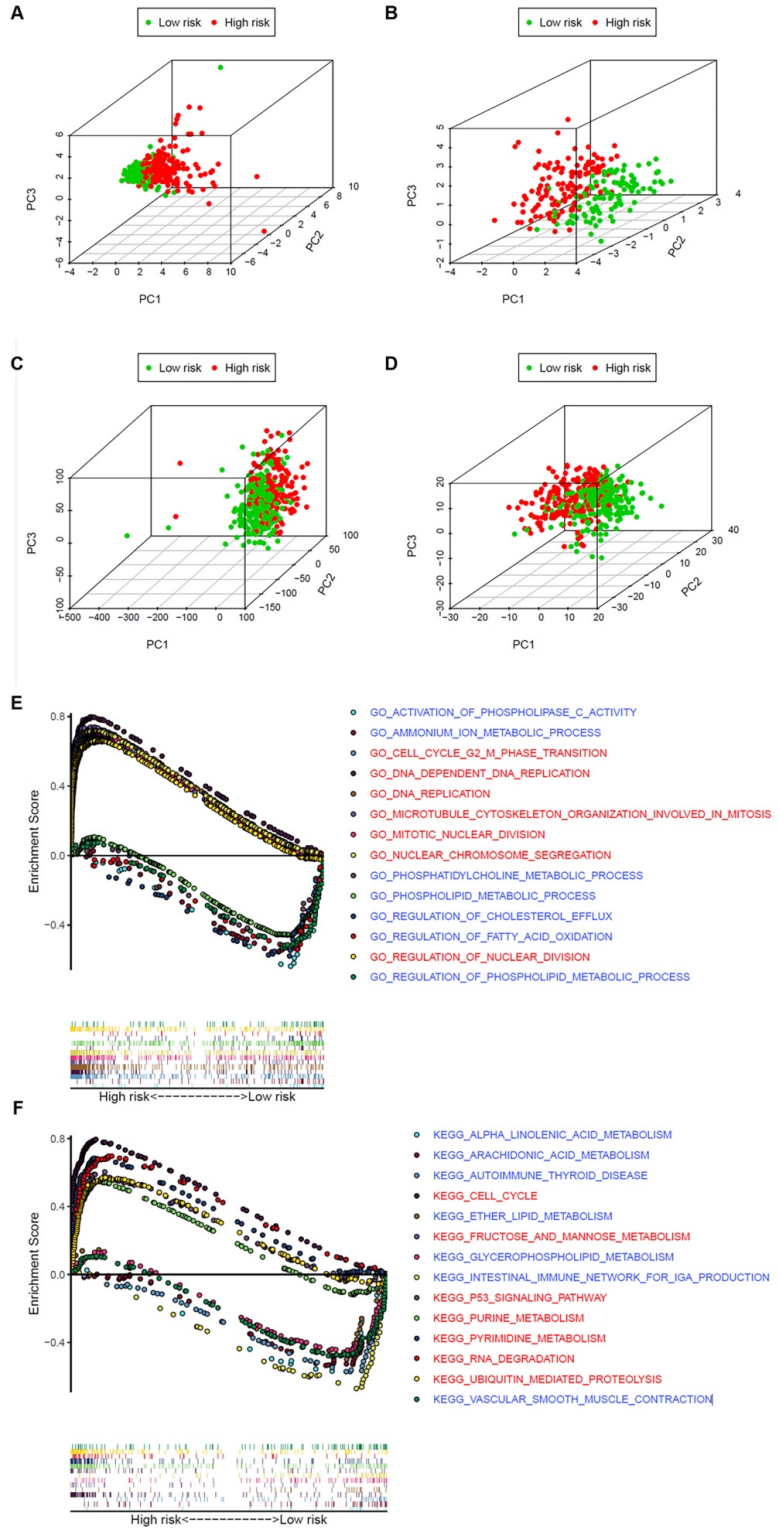
The high‐ and low‐risk groups showed different distribution patterns and gene-set enrichment analysis. **A**. PCA of the high- and low-risk groups based on the 8 risk genes in the training set. **B**. PCA of the high- and low-risk groups based on 8 risk genes in the validation set. **C**. PCA of the high- and low-risk groups based on the whole genome set. **D**. PCA of the high- and low-risk groups based on the whole metabolism-related genes. **E**. KEGG and **F**. GO analysis by GSEA. The red font represents high-risk, while blue fonts represent low-risk.

To explore whether the LUAD patients could be distinguished properly based on our prognosis model, PCA was utilized to explore the distinct distribution modes between the 2 groups (lows and high-risk). According to the risk genes, these 2 groups were divided into 2 aspects in the training and validation set (Figure 7A and B). Based on either the whole-genome sets or the whole metabolism-related genes, high- and low-risk groups showed significant separation distribution (Figure 7C and D). The GSEA further validated the functional annotations and found that the high-risk group was concentrated in mitosis and proliferation, while the low-risk group was mainly correlated with immunity and metabolism (Figure 7E and F), which was accordant with the preceding consequence of TIMER. Moreover, our results suggested that patients in the high-risk group were more likely to be male and at an advanced clinical stage (Fisher's exact test, p < 0.05, Table 1).

### WGCNA

Based on the enrichment values, patients were classified into 3 groups: high-, moderate-, and low-immunity groups (Figure S4A). The ESTIMATE algorithm was utilized to calculate the immune values. The immune scores of the high-, middle- and low-immunity groups decreased in turn (Figure S4B).

To build a co-expression network of metabolism- and immune-related genes, we used WGCNA analysis. The power of β = 5 (scale-free R2 = 0.91) was chosen as the soft threshold parameter (Figure S5A), ensuring a network of no scale. The module eigengene (ME) was then used to represent the whole gene expression levels of the corresponding modules and to investigate the co-expression resemblance of these modules (Figure S5B). Five modules were recognized by the average linkage hierarchical clustering (Figure S5C). The correlations between ME and the immune scores were calculated to explore the relationship between gene modules and immune characteristics. The yellow module had the most powerful correlation with the immune scores. These 5 modules were divided into 2 clusters, among which the yellow module was the closest to the immune scores (Figure S5D).

**Figure 8:**
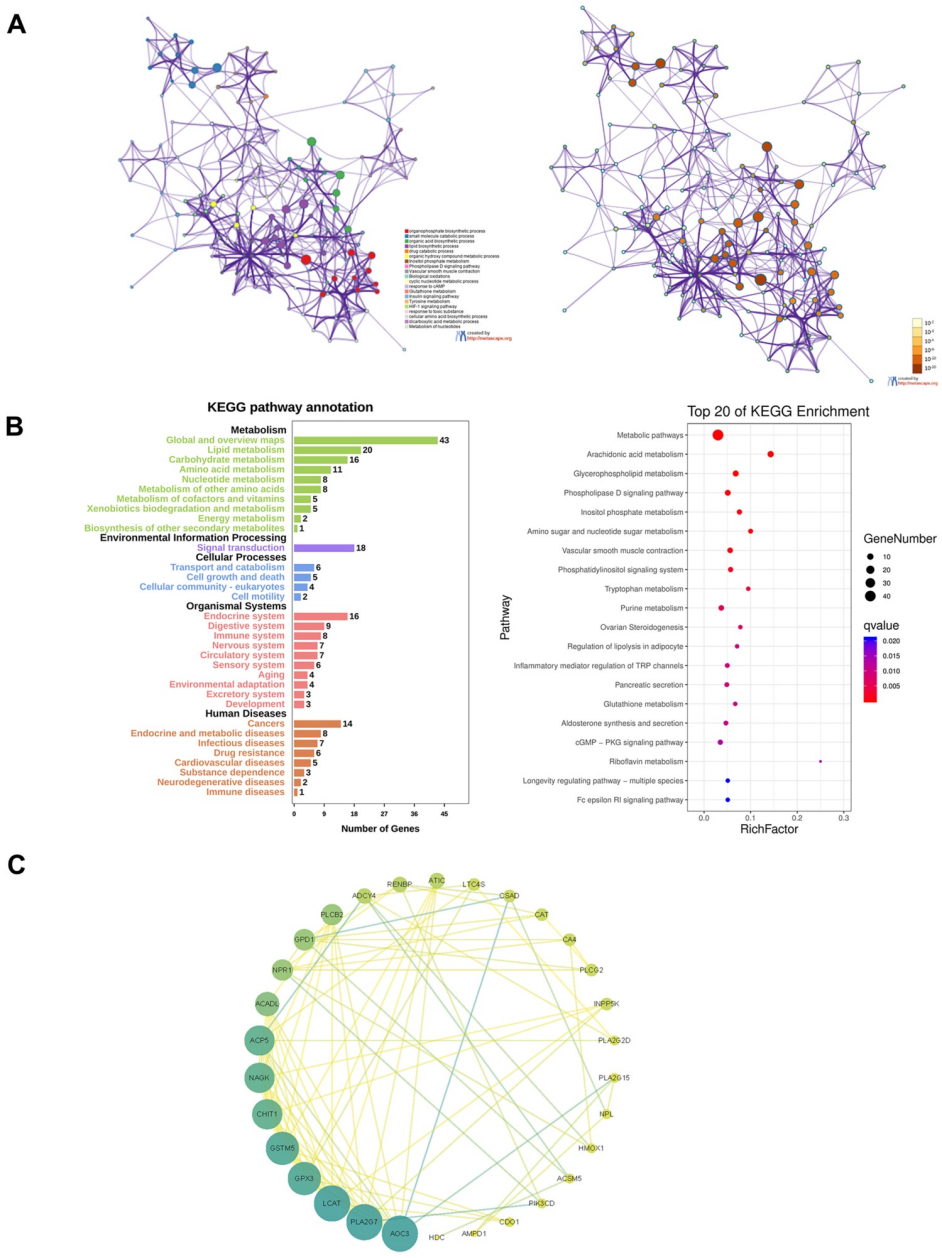
Functional enrichment analysis of the yellow module and screening of hub genes. **A**. GO analysis. **B**. KEGG analysis. **C**. The regulation network diagram according to the topological overlap between the genes.

All the genes of the yellow module were extracted for functional enrichment analysis. GO and KEGG enrichment analysis showed the genes in the yellow module were not only related to metabolism but also immune system (Figure 8A and B). To screen out the hub genes in the yellow module, we calculated the topological overlap between these genes. The regulation network diagram was drawn using Cytoscape. AOC3, PLA2G7, LCAT, GPX3, and GSTM5 were the top 5 hub genes in the yellow module (Figure 8C).

## Discussion

The importance of differential genes in cancer deterioration and immunotherapy has been recognized, but the overall genome-wide analysis is still to be investigated to explore the molecular mechanism and clinical significance. Our studies revealed the effects of metabolism-related genes on LUAD clinical significance and elucidated the molecular characteristics. A total of 27 metabolism-related genes were significantly related to the occurrence and development of LUAD, which might be valuable clinical indicators. Personalized metabolism-related prognostic characteristics on the basis of selective metabolism-related genes could be used to evaluate potential clinical outcomes and measure immune cell infiltration.

To establish a suitable and simple scheme to observe the metabolic status of LUAD patients and imply clinical outcomes, we built a metabolism-based prognostic index. With the consequences of LASSO regression analysis, the prognostic indexes based on 8 metabolism-related genes (TYMS, ALDH2, PKM, GNPNAT1, LDHA, ENTPD2, NT5E, MAOB) were established. Patients with high-risk values have a bad prognosis, whose survival time was shortened with increased risk values. Moreover, univariate COX and multivariate COX regression analysis illustrated that the prognostic signature based on these metabolism-related genes might be applied as independent prognostic factors. We also constructed a nomograph composed of metabolism-related genes and other clinical factors to predict the OS. Our studies suggested that metabolism-related genes could be used as prognostic markers and indexes of metabolic status.

The mechanism and function of ENTPD2 in lung cancer were not reported previously. The other 7 metabolism-related genes TYMS, ALDH2, PKM, GNPNAT1, LDHA, NT5E and MAOB have been reported. TYMS was involved in gene replication, which was highly related to the poor prognosis of NSCLC. Studies found that repressed TYMS expression improved the sensitivity of lung cancer cells to pemetrexed 21. The main function of ALDH2 was to detoxify acetaldehyde (ACE) into non-toxic acetic acid 22. Li et al. found that inhibited ALDH expression not only led to poor prognosis of LUAD but also enhanced tumor cell proliferation, stemness, and migration, which was related to the increase of DNA damage caused by ACE accumulation 23.

PKM was reported to involve in the process of glycolysis. Inhibited PKM2 expression decreased lung cancer cell proliferation 24. PKM2 could form complexes with FGFR1 and RACK1 to participate in the occurrence and development of lung cancer 25. Zhao et al. found that Abraxane, which was an albumin-bound nanoparticle drug for treating NSCLC, repressed GNPNAT1 expression, resulting in inhibited tumor cell proliferation 26. LDHA was also involved in glycolysis, catalyzing the oxidation of lactic acid to pyruvate. LDHA was upregulated in most tumors and related to the poor prognosis of cancer 13. Li et al. found that the radiosensitivity of NSCLC was enhanced by inhibiting LDHA 27. It was reported that NT5E was overexpressed in NSCLC and inhibited by miR-30a-5p, involving in NSCLC cell migration, invasion, and proliferation 28. Son et al. found that MAOB was repressed by Danshensu, resulting in the inhibited NF-κB signaling 29. Although the function of ENTPD2 in NSCLC was not reported, it was proved to cause immune escape via inhibiting myeloid-derived suppressor cell (MDSC) differentiation in liver carcinoma 30.

Impressive progress in comprehending tumorigenesis and clinical treatment techniques was achieved in recent decades, but many aspects of the molecular mechanism related to LUAD metabolic are still unclear. Our studies focused on the changes in metabolic and genomic profiles to reveal the relationship between metabolic status and these profiles.

Due to the rapid growth of tumors, the imbalance between oxygen demand and supply led to tumor hypoxia. Hypoxia of tumor cells induces tumor cells to release immunosuppressive factors, resulting in immune escape 31, 32. Meanwhile, the lack of energy and nutrients of immune cells caused by the rapid growth of tumors might also be the reason for immunosuppression 33. The Warburg effect of tumor tissue could produce a considerable lactic acid, which was reported to induce M2 macrophage polarization 34. M2 macrophages facilitated tumor developing, immune escape, and invasion 35-37.

Based on the 8 metabolism-related genes in LUAD, this prognostic indicator showed clinically satisfactory feasibility. B cells, CD4+ T cells, dendritic cells and macrophages were inversely related to the risk scores. Previous studies showed that the proportion of antibodies was related to the density of follicular B cells 38. Follicular B cells and tumor-infiltrating plasma cells were associated with better prognosis of lung cancer patients 39. CD4+ T cells emit multifarious cytokines with direct effects to activate other immune cells 40, 41. Tumor-infiltrating CD4+ T cells were also associated with better prognosis of NSCLC patients 42. As antigen-presenting cells, dendritic cells played a significant role in adaptive immune response, but their roles of antigen recognition, processing and presentation were usually destroyed or blocked during tumor development 43-45. Kimura et al. found that the adoptive transfer of autologous activated killer T cells and dendritic cells increased the OS of lung cancer patients and the proportion of CD8+/CD4+ T cells 46. Tumor-associated macrophages originated from peripheral mononuclear cells, whose tumor-promoting functions contained backing tumor-related angiogenesis and facilitating cancer cell invasion, migration, and vascular migration 37, 47, 48. M1 macrophages located in islets of tumor cells were usually associated with better prognosis, while more abundant M2 macrophages in tumor stroma were correlated with poor prognosis 49. With the increase of risk values, the numbers of B cells, CD4+ T cells, dendritic cells, and macrophage decreased, resulting in poor prognosis of LUAD patients. Our studies suggested that metabolism-related genes had the capacity to be predictors of immune cell infiltration.

In this study, we screened out genes related to metabolism and immunity by the WGCNA algorithm and obtained a total of 5 gene modules. Subsequently, we analyzed the functional enrichment of the gene module highly related to immune scores. The hub gene of this module were identified with Cytoscape. Except for GPX3 and GSTM5, there was no report on the other hub genes in LUAD 50, 51. Our results combined metabolism with immunity to identify new therapeutic targets in LUAD.

Our current study had some shortcomings, which should be taken into account when explaining our consequences. First, transcriptome analysis could only reflect certain aspects of the immune state, but not global changes. Secondly, the verification with another independent queue was lacked. At the last, our results also required validation of in vivo and in vitro experiments.

The correlation between proteomics, metabolomics, and immunogenomics ought to be investigated to characterize the overall immunological alternations in LUAD. Importantly, the latent correlation between the precancerous lesions and the disrupted metabolomic genome was to be further investigated. We predicted that this prognostic feature might have important clinical significance. Our studies offered novel understanding of the development of new therapeutic targets in LUAD.

## Conclusions

Based on gene sets downloaded from the TCGA database, we utilized LASSO and univariate COX regression analysis to screen metabolism-related genes correlated with the prognosis of LUAD patients. A prediction model was constructed based on 8 metabolism-related genes (TYMS, ALDH2, PKM, GNPNAT1, LDHA, ENTPD2, NT5E, MAOB). This model well predicted immune cell infiltration in LUAD. Our study provided a potential model and biomarkers for further metabolism-related work and personalized medicine for LUAD treatment.

## Supplementary Material

Supplementary figures.

## Figures and Tables

**Table 1:** Clinicopathologic characteristics of patients in different risk groups.

Characteristics	Whole cohort	Low risk	High risk	*P*
Case	454	223	222	
Age				0.8409
≤ 60	152 (33.5)	76 (34.1)	73 (32.9)	
> 60	302 (66.5)	147 (65.9)	149 (67.1)	
Gender				0.0173
Female	248 (54.6)	135 (60.5)	109 (49.1)	
Male	206 (45.4)	88 (39.5)	113 (50.9)	
Stage				0.002548
Stage I	243 (53.5)	140 (62.8)	98 (44.1)	
Stage Ⅱ	105 (23.1)	45 (20.2)	57 (25.6)	
Stage Ⅲ	74 (16.3)	26 (11.7)	47 (21.2)	
Stage Ⅳ	24 (5.3)	7 (3.1)	17 (7.7)	
T (Tumor)				
T1	156 (34.4)	96 (43.0)	56 (25.2)	0.000415
T2	240 (52.9)	103 (46.2)	132 (59.5)	
T3	37 (8.1)	17 (7.6)	20 (9.0)	
T4	18 (4.0)	5 (2.2)	13 (5.9)	
N (Lymph Node)				0.005335
N0	291 (64.1)	157 (70.4)	129 (58.1)	
N1	86 (18.9)	36 (16.1)	47 (21.2)	
N2	64 (14.1)	22 (9.9)	41 (18.5)	
N3	2 (0.4)	0 (0)	2 (0.9)	
M (Metastasis)				0.03075
M0	305 (67.2)	148 (66.4)	148 (66.7)	
M1	23 (5.1)	6 (2.7)	17 (7.7)	

**Table 2:** General characteristics of LUAD metabolism-related genes.

ID	coef	HR	HR.95L	HR.95H	pvalue	logFC	FDR
TYMS	0.012119	1.02969	1.014094	1.045526	0.000172	1.531233	5.49E-26
ALDH2	-0.00056	0.978645	0.964734	0.992756	0.003123	-1.07487	8.27E-23
PKM	0.000501	1.004606	1.002274	1.006944	0.000106	1.342386	1.17E-23
GNPNAT1	0.022241	1.046756	1.029999	1.063786	2.86E-08	1.208602	4.34E-26
LDHA	0.002514	1.005066	1.003374	1.006762	4.20E-09	1.923462	4.44E-27
ENTPD2	0.061361	1.124384	1.065047	1.187026	2.25E-05	1.121139	1.45E-10
NT5E	0.000165	1.010262	1.00277	1.017811	0.007184	1.921271	5.87E-09
MAOB	-0.00403	0.966209	0.935137	0.998312	0.03928	-1.20903	1.17E-23

**Table 3:** Relationships between the expressions of the metabolism-related genes and the clinicopathological factors in LUAD.

Gene symbol	Age (≥65/<65)		Gender (male / female)		Stage (I & II / III & IV)		T stage (T1-T2 / T3-T4)		N stage (N0 / N1-3)	
	t	P	t	P	t	P	t	P	t	P
TYMS	-0.048	0.962	-1.628	0.105	-1.534	0.128	1.242	0.218	-0.271	0.787
ALDH2	-1.829	0.069	-0.762	0.447	1.032	0.304	0.348	0.729	1.326	0.186
PKM	-0.081	0.935	-0.544	0.587	-2.908	0.004	-1.822	0.075	-3.051	0.003
GNPNAT1	0.517	0.605	-2.552	0.011	-2.357	0.021	-1.415	0.165	-1.418	0.157
LDHA	0.72	0.472	-0.976	0.33	-2.647	0.01	-1.884	0.066	-2.508	0.013
ENTPD2	0.581	0.562	0.236	0.813	-1.402	0.164	-0.896	0.375	-0.463	0.644
NT5E	-0.702	0.483	1.995	0.047	-1.282	0.203	-1.285	0.205	-0.434	0.665
MAOB	-1.79	0.075	0.629	0.53	3.918	1.14E-04	1.99	0.05	1.09	0.277
riskScore	0.916	0.361	-2.006	0.046	-3.761	2.99E-04	-2.137	0.038	-2.503	0.013

## Abbreviations

LUAD: lung adenocarcinoma
TME: tumor microenvironment
NSCLC: Non-small cell lung cancer
OS: overall survival
ROS: reactive oxygen species
LDH-A: lactate dehydrogenase-A
FDR: false discovery rate
LASSO: least absolute shrinkage and selection operator
AUC: area under the curve
