# Supplementary material for: Establishment of the Prognostic Index Reflecting Tumor Immune Microenvironment of Lung Adenocarcinoma Based on Metabolism-Related Genes
Source: J Cancer. 2020 Oct 18;11(24):7101–15. doi: 10.7150/jca.49266 (PMC7646164; doi:10.7150/jca.49266)
Supplement: Supplementary file 1 — Supplementary figures. [file jcav11p7101s1.pdf]

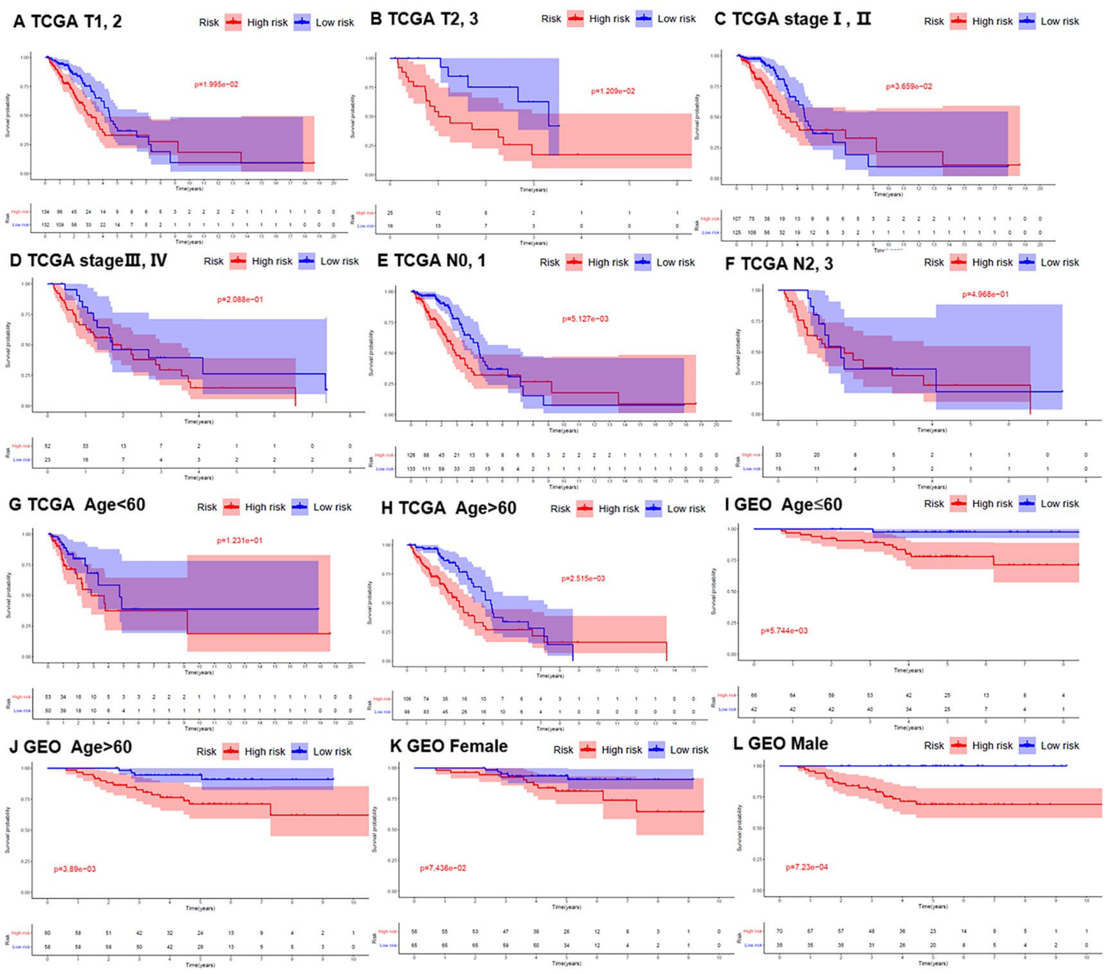

Figure S1**.** Subgroup analysis in the training set and validation set. Kaplan–Meier survival for the OS in subgroups stratified by **A**. T 1, 2, **B**. T 3, 4, **C**. stage I, II, **D**. stage III IV, **E**. N 0, 1, **F**. N 2, 3, **G**. Age ≤ 60 and **H**. Age > 60 in the training set, and **I**. Age < 60, **J**. Age > 60, **K**. Female and **L**. Male in the validation set.

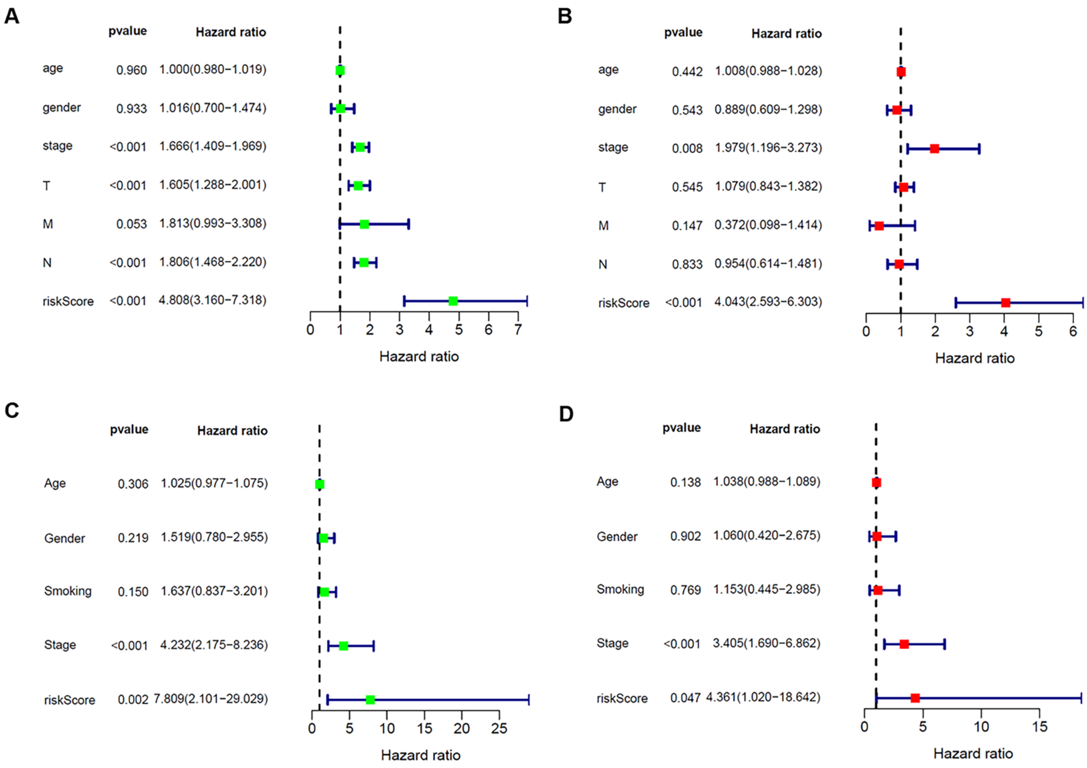

Figure S2**.** Independent prognostic analysis. **A**. Univariate and **B**. Multivariate COX regression analysis of LUAD in the training set. **C**. Univariate and **D**. Multivariate COX regression analysis of LUAD in the validation set.

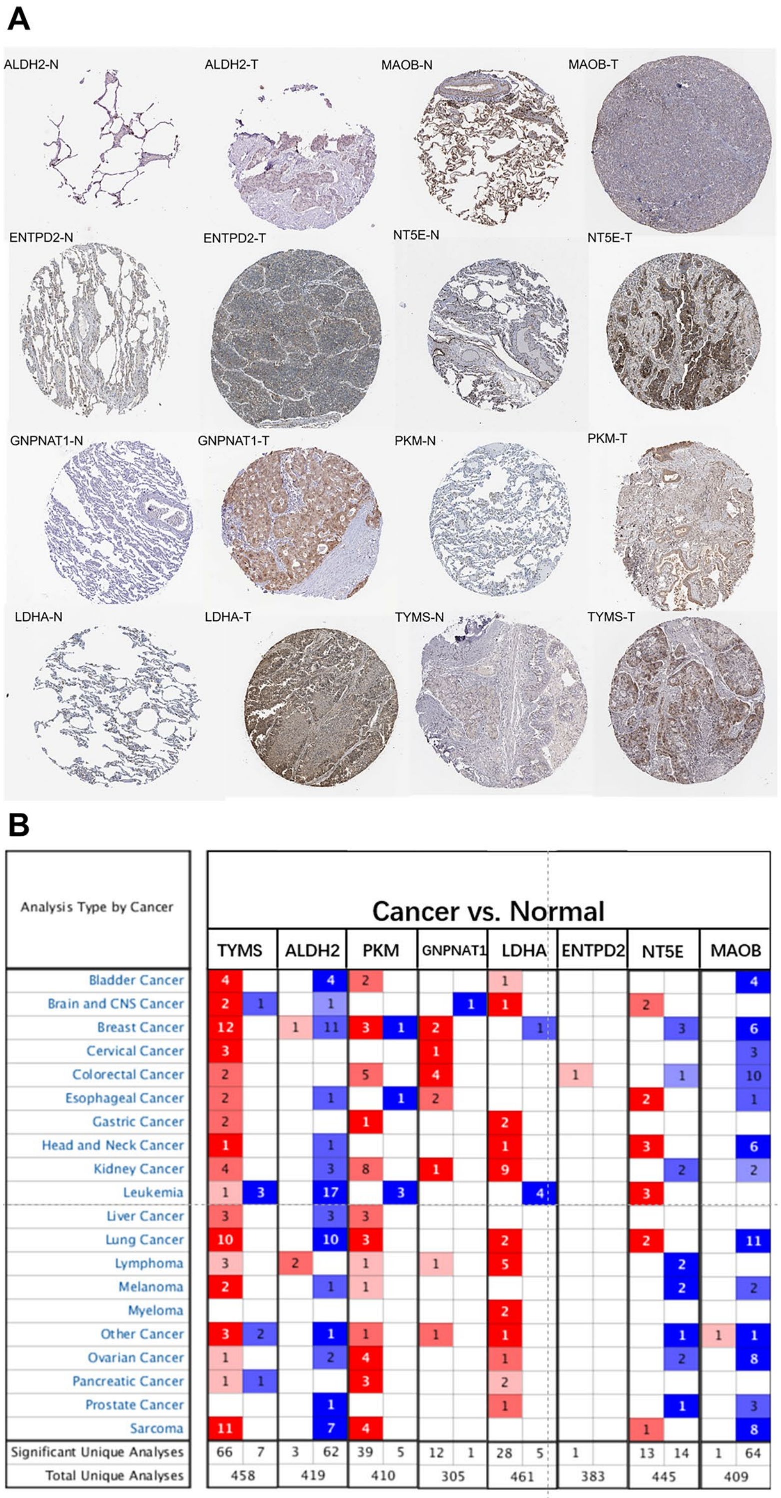

Figure S3**.** Validation of the metabolic-related genes. **A**. Immunohistochemistry of the metabolism-related genes based on the Human Protein Atlas. The mRNA expression patterns of metabolism-related genes in overall cancers. **B**. The differences in mRNA expression between tumors and normal tissues were analyzed by the Oncomine database.

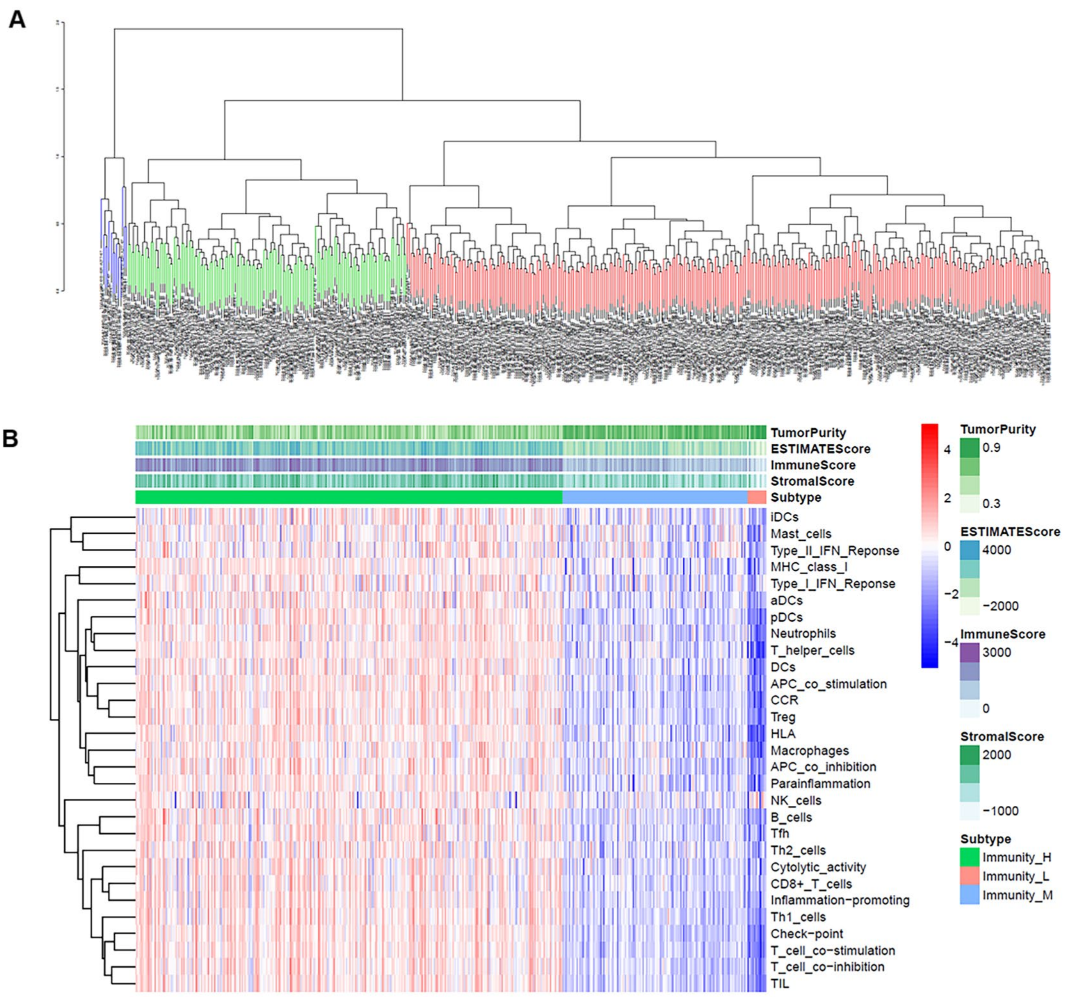

Figure S4**.** Single-sample Gene Set Enrichment Analysis (ssGSEA). **A**. Hierarchical clustering of LUAD based on the enrichment scores. **B**. Heatmap showing the correlation of transcriptome expression with diverse immune cell types, functions, and pathways.

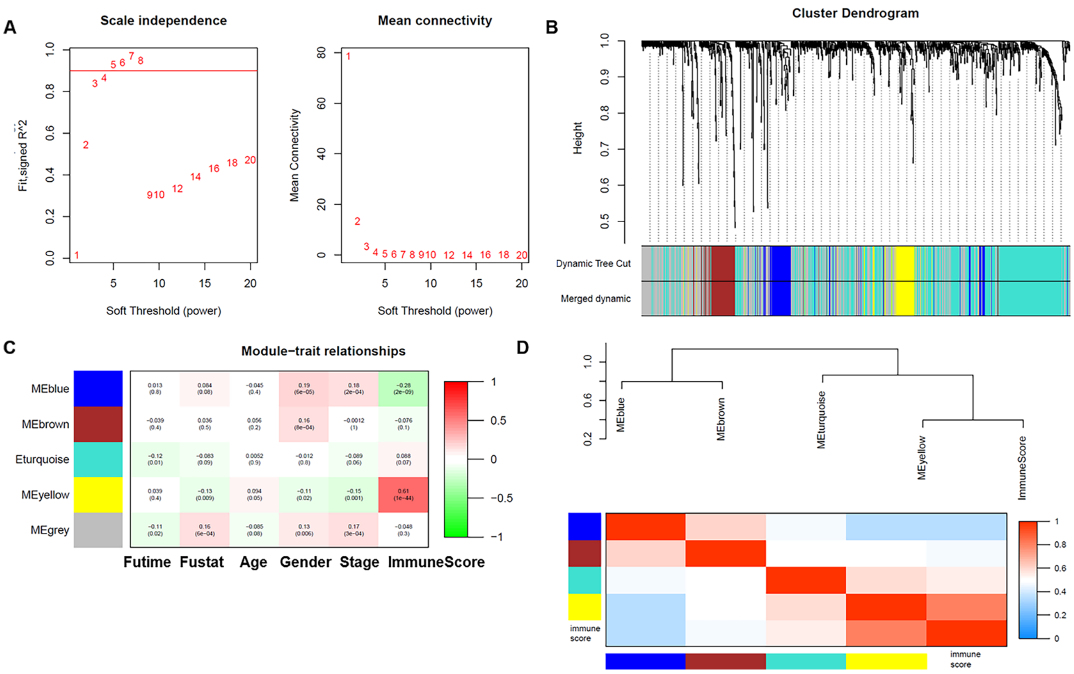

Figure S5. WGCNA analysis. **A**. Analysis of the scale-free fit index for various soft-thresholding powers (β) and the mean connectivity for various soft-thresholding powers. Clustering dendrograms of metabolic-related genes. **B**. Each piece of the leaves on the cluster dendrogram corresponds to a gene. **C**. Correlation between the module eigengenes and clinical traits of lung adenocarcinoma. **D**. Hierarchical clustering and heatmap of the gene module.
